# Predicting suicide probability in young adults: contributions of vulnerable narcissism, grudge, and demographic variables

**DOI:** 10.3389/fpsyg.2026.1821283

**Published:** 2026-05-29

**Authors:** Gülin Yazıcı Çelebi

**Affiliations:** Department of Psychology, Faculty of Literature, Gümüşhane University, Gümüshane, Türkiye

**Keywords:** gender, grudge, narcissim, suicide, young adults

## Abstract

Suicide is a significant public health problem worldwide. The aim of this study is to examine the potential effects of levels of vulnerable narcissism and grudge on the likelihood of suicide. In addition to these variables, the potential effects of some demographic variables (gender, marital status, age, socioeconomic status, education level, perceived religiosity, number of siblings) were also investigated. A convenience sampling method was used to determine the study group. The study group consisted of a total of 500 individuals; 368 (73.6%) were women and 132 (26.4%) were men. The mean age of women was 26.31 (SD = 9.60, min-max = 18–64), the mean age of men was 29.01 (SD = 10.76, min-max = 18–55), and the mean age of the entire sample was 26.84 (SD = 9.88, min-max = 18–64). The Suicide Probability Scale, Vulnerable Narcissism Scale, Grudge Scale, and Personal Information Form were used as data collection tools in the study. Pearson correlation coefficient was used to examine the relationships between variables in the analysis of the data. Multiple linear regression analysis was conducted to examine the extent to which vulnerable narcissism, grudge, and sociodemographic characteristics are associated with suicide probability. The regression model showed that the independent variables significantly predicted the probability of suicide. According to the results obtained, the independent variables explained 38% of the variation in the probability of suicide {[*R* = 0.619, *R*^2^ = 0.383, *F*_(8, 499)_ = 38.12, *p* < 0.0001]}, and vulnerable narcissism and grudge are positive predictors of the probability of suicide. Vulnerable narcissism (β = 0.34, *p* < 0.001) and grudge (β = 0.22, *p* < 0.001) are positive predictors of the probability of suicide. Age (β = −0.23, *p* < 0.001), education level (β = −0.08, *p* = 0.003), and SEL (β = −0.19, *p* < 0.001) are significant variables that negatively predict the probability of suicide. Based on the results, it can be said individuals with high levels of narcissism and grudge have a higher probability of suicide, and that some socio-demographic characteristics may influence these relationships. The findings are discussed in light of the literature.

## Introduction

Suicide can be defined as individuals' intentional or voluntary ending of their own life and it has become an important problem due to the increasing number of people who commit suicide each day. The gravity of the situation becomes clearer when the number of attempted suicides is taken into

account in addition to the number of completed suicides. World Health Organization predicts that by 2030, the number of people who commit suicide will exceed one million (World Health Organization, 2019). Suicide has attracted the attention of humanity in every period and despite many prevention policies, it is still one of the major causes of death. It has many psychological, sociological, economic, religious, etc. dimensions that differ epidemiologically and etiologically depending on the society and culture. Although different disciplines conduct different studies, the common question is “what are the factors that lead individuals to suicide?” The aim in most of the studies is to identify early signs and risk factors and to prevent suicides. These findings are of great importance for prevention and protection studies. In the literature, theories about suicide draw attention as much as research. From the past to the present, a large number of scientists have tried to make sense of the psychological factors that have an impact on suicide and thus various theories have emerged. Durkheim was the first to define the concept of suicide, and since then it has been considered as a social problem. While Freud (1916) associated suicide with the loss of love object and depression, Menninger expressed it as self-directed aggression (Odag, 2002). There are also theories called second generation theories in addition to traditional theories explaining suicide. The basis of the present study is the theory called Fluid Vulnerability Theory, which is one of the second generation theories. The theory put forward by Rudd (2006) is considered to be among cognitive theories. Unlike many theories in the literature, it focuses on risk factors rather than treatment. Fluid Vulnerability Theory considers suicide as a dynamic structure. According to the theory, suicide risk comprises both dynamic and stable components. The dynamic characteristics are responsive to environmental and individual factors, while the stable characteristics remain relatively constant and resistant to change. Suicidal behavior emerges from the interaction between these acute, dynamic risk factors and the more basic, enduring risk processes. The theory emphasizes two groups of risk factors related to suicide: basic and acute. In the FVT, the basic risk consists of relatively stable risk factors called predispositions for the suicidal thoughts and behaviors of the individual. Basic risk is specific for the individual and consists of factors related to past history and dispositional factors, which may include both suicide-specific cognitions and cognitive processes related to suicidal stimuli. Acute risk is activation of suicidal mode. Reactivity, cognitive flexibility and perceived burden are examples of dispositional factors. These dispositional factors are similar to personal characteristics and they are stable over time. An individual may commit suicide acutely when exposed to stress that is strong enough to go beyond the basic risk threshold. This process is called “activation” (Moscardini et al., 2020; Bulut and Demirbaş, 2021). According to the Theory, while some persistent personality traits may reflect fundamental vulnerability, situational or cognitive-emotional processes can act as acute triggering factors (Rudd, 2006). Accordingly, in this study, vulnerable narcissism is conceptualized as a potential fundamental vulnerability factor, while resentment is considered as a proximate cognitive-emotional trigger associated with increased suicidal probability.

Research in the literature on the risk factors for suicidal ideation include depression (Fonseca-Pedrero et al., 2022, 2024), mood disorders (Beautrais, 2002), personality disorders (May et al., 2012), and emotional dysregulation (Fonseca-Pedrero et al., 2024). The present study examines the association of narcissism and grudge levels with suicide probability. Although there are many research findings related to the association of suicide probability with self-esteem, there are limited number of studies on narcissism, which is considered as a different dimension of this. It is seen that borderline personality disorder is frequently focused on as personality disorder. For this reason, the study included vulnerable narcissism as a variable.

### Vulnerable narcissism

The origins of the concept of narcissism go back to Narcissus, a figure in Greek mythology who fell in love with his own reflection in a pool of water. There are theories that work on this issue, such as psychoanalytic theory, object relations theory and self-psychology theory. When the literature is reviewed, there are studies defining narcissism as a condition characterized by excessive self-importance, ignoring others and experiencing problems in interpersonal relationships as a result of this approach (Kealy and Rasmussen, 2012). DSM-III included narcissism as a pathology, while DSM-V shaped its final form. DSM-V included and discussed narcissistic personality disorder as one of the 12 personality disorders. There are two dimensions of narcissism as grandiose narcissism and vulnerable narcissism. Studies on grandiose narcissism and vulnerable narcissism mostly draw attention to their opposite characteristics. Features such as being excessively demanding, the desire to attract attention, exhibitionism, feeling superior, envy, arrogance, ignoring the needs of others and low anxiety stand out in grandiose narcissism. In contrast to grandiose narcissism, excessive humility, hypersensitivity to criticism, timidity, constant stress and grandiose expectations are predominant in vulnerable narcissism (Ronningstam et al., 2018). In general, both types of individuals believe that they are extremely superior and unique. However, while grandiose narcissists may show aggressive antisocial behaviors to protect this perception and to gain the admiration of others, vulnerable narcissist may feel ashamed of these grandiose thoughts, try to mask these with excessive modesty and prefer to distance themselves from social relationships with the anxiety of rejection. In other words, while the grandiose fantasies they have are common, the way they handle these and reflect these in life are different. In vulnerable narcissism, individuals are highly sensitive to being criticized and evaluated and they avoid environments where this possibility appears. This tendency makes them more vulnerable. A study found that vulnerable narcissists had much longer duration of focusing on negative stimuli than grandiose narcissists (Krusemark et al., 2015). Another study associated this negative affect in vulnerable narcissists with high level of depression and anxiety (Rathvon and Holmstrom, 1996). Individuals may show narcissistic personality traits, even if not at the level of personality disorder. A 15-year-long follow-up study concluded that individuals with narcissistic traits were significantly more likely to die as a result of suicide when compared with individuals who did not have these traits (Stone, 1989). Another study reported depressive older individuals with narcissistic traits to be at greater risk for suicide (Heisel et al., 2007; Sher, 2016). Based on this information, the present study included vulnerable narcissistic traits as a variable to examine how they affect the likelihood of suicide in individuals. Ronningstam and Maltsberger (1998) suggested that one of the underlying causes of suicidal behaviors in individuals with narcissistic personality disorder may be a vengeful act against narcissistic damage and the desire to eliminate a defective self. This focus on revenge and unforgiveness led to the consideration of grudge as a potential variable associated with suicide probability, and thus it was included as a variable in the study.

### Grudge

Grudge is defined as contempt, hatred, an intense desire to harm and take revenge and grudge caused by hostile feelings (Jensen, 2010). According to Tarhan (2019), the desire to make the other party pay a price underlies the feeling of hatred, which means hidden enmity. During the process, the individual does not care about the price s/he will pay. There are studies discussing grudge from different perspectives. Scientists who discuss grudge in terms of psychopathology have stated that it is closely related to antisocial personality disorder and borderline personality disorder, making it possible to use it as a criterion during the diagnosis process (Velotti et al., 2017). According to another view, grudge is motivated by the basic feeling of anger and this situation is universal (Marlowe et al., 2011). Studies conducted indicate that individuals with high levels of grudge show a high tendency to behave hostile and low tendency to regret this, while they have more difficulty in empathizing than other individuals (Ewing et al., 2016). A study reported that grudge can be a predictor of violence (Rogier et al., 2020). Therefore, considering its potential impact on individuals' suicidal tendencies, the feeling of grudge was included as a variable in the present study. Considering that making the other party pay a price underlies the feeling of grudge, it may create the thought of punishing someone with being gone and the remorse of consciousness, associating this situation with suicide. The present study also examined how suicide probability is associated with the variables of sex, age, marital status, educational status, socio-economic level, perceived grudge and the number of siblings. It can be seen that there are studies in literature which show that these variables are associated with suicide probability. The main purpose of the present study is to investigate the associations between various variables and suicide probability.

Although narcissistic personality disorders are not very common, narcissistic personality traits are very common in a large number of individuals. Therefore, examining the relationship between narcissistic traits and suicide will contribute to the literature. Grudge is a trait that individuals have at varying levels. Addressing these will benefit the literature and be guiding for studies to prevent suicide. There are no studies in literature which have discussed vulnerable narcissism, grudge and suicide probability together. It is possible to prevent suicide and studies on the issue will provide important contributions in terms of determining risk factors, protective factors and early signs. Understanding the related factors is important in terms of providing information to suicide prevention strategies and programs and developing more detailed and effective interventions.

## Method

### Study group

The method used for determining study group was convenience sampling method. This method enables researchers to gather data from participants who are more accessible and cost-effective in terms of time, financial resources, and overall accessibility (Creswell, 2014). A power analysis was conducted to determine the sample size. For this purpose, G^*^Power 3.1 software was used for a multiple regression model including nine variables thought to be associated with the probability of suicide (Faul et al., 2009). According to the preliminary power analysis results, with an alpha level of 0.05, statistical power of 0.95 (1–β), and a moderate effect size (*f*^2^ = 0.15), the minimum sample size was determined to be 180. The choice of a moderate effect size (*f*^2^ = 0.15) is based on the classifications recommended for multiple regression analyses in social and behavioral sciences (Cohen, 1988; Balkin and Sheperis, 2011). Nine variables were included in the analysis within the scope of the research model. These variables were structured as follows: (suicide probability) as the dependent variable, and fragile narcissism, grudge, gender, age, marital status, education level, socioeconomic status, and level of religiosity as independent variables. The resulting sample size in the study consists of a total of 500 individuals, 368 (73.6%) women and 132 (26.4%) men. The sample shows an unbalanced gender distribution. This may be related to the preference for voluntary participation and convenience sampling in the data collection process. The literature indicates that higher representation of female participants is common in social and behavioral sciences, particularly in studies based on voluntary participation (Smith, 2008). Mean age of women was 26.3 (SD = 9.60, min-max = 18–64), mean age of men was 29.01 (SD = 10.76, min-max = 18–55) and mean age of the whole sample was 26.84 (SD = 9.88, min-max = 18–64). Table 1 shows the demographic information of the participants in detail.

**Table 1:** Demographic variables.

Demographic variables of participants	*F*	%
Sex		
Female	368	73.6
Male	132	26.4
Marital status		
Single	365	73
Married	135	27
Education level		
High school and lower	66	13.2
Undergraduate	392	78.4
Post graduate	42	8.4
Socio-economic level		
Low	66	13.2
Moderate	394	78.8
High	40	7.8

Table 1 shows that a great majority of the participants consist of individuals who are female, single, with an undergraduate level of education and moderate socio-economic level.

### Data collection tools

#### Vulnerable narcissism scale

Hendin and Cheek (1997) developed this scale to determine levels of vulnerable narcissism and Sengül et al. (2015) adapted the scale into Turkish. The 8-question scale has a 5-Likert design. The Vulnerable Narcissism Scale has a Cronbach's alpha coefficient of 0.66, and the scale has been adapted as a single dimension, hypersensitivity. Scores above average indicate the presence of vulnerable narcissism. In their study, Sengül et al. (2015) found mean scores as 22.29 for female participants and as 23.46 for male participants. The confirmatory factor analysis performed for our study tested the single-factor structure of the scale. The obtained fit values (χ^2^ = 41.97; Sd = 17; χ^2^/sd = 2.47; *p* < 0.001; RMSEA = 0.05; SRMR = 0.04; CFI = 0.94; TLI = 0.92) showed that the single factor of the scale had an acceptable level of fit with the research data (Hair et al., 2010). The factor loadings of the scale items ranged from 0.39 to 0.65. Previous adaptation studies reported a relatively modest reliability coefficient for the vulnerable narcissism scale (α ≈ 0.66). However, in the present study, the internal consistency coefficient was found to be satisfactory (α = 0.75), indicating good reliability (Hair et al., 2019). This difference may be related to sample characteristics or contextual factors.

### Suicide probability scale

Cull and Gill (1988) developed the Suicide Probability Scale (SPS) to identify adolescents and adults at risk of suicide attempts, and Atli et al. (2009) adapted the scale into Turkish. The 36-item, 4-point Likert-type scale is assessed using four subscales: hopelessness, hostility, suicidal ideation, and negative self-evaluation, with 9 items reverse-coded. Possible scores range from 36 to 144, with higher scores indicating a higher probability of suicide. The internal consistency reliability calculated for this research is 0.89. A four-factor structure was tested using confirmatory factor analysis. The obtained fit values (χ^2^ = 1729.38; Sd = 576; χ^2^/sd = 3.00; *p* < 0.001; RMSEA = 0.06; SRMR = 0.05; CFI = 0.95; TLI = 0.94) showed an acceptable level of fit for the four constructs of the scale (Hair et al., 2010). The factor loadings of the items ranged from 0.35 to 0.79. Alpha coefficients were found to be between 0.77 and 0.90 for the factors and 0.88 for the overall scale.

### Grudge mood scale

Ersanli and Göksü (2018) developed the 21-item, 4-subscale Grudge Mood Scale (GMS). The scale is a 5-point Likert type scale. Confirmatory factor analysis was performed to test the structural validity of the scale for this research. The obtained fit values Sχ^2^ = 487.42; Sd = 156; χ^2^/sd = 3.12; *p* < 0.001; RMSEA = 0.06; SRMR = 0.05; CFI = 0.95; TLI = 0.93) showed an acceptable level of fit (Hair et al., 2010). The factor loadings of the scale items ranged from 0.36 to 0.82. The alpha coefficient calculated for the scale was 0.92.

### Demographic information form

The form asked about gender, age, marital status, education level, socioeconomic status, and level of religiosity. Level of religiousness was assessed using a single-item self-report measure, in which participants rated their level of religiousness on a scale from 1 to 10. Single-item measures are commonly used in large-scale and survey-based research when the construct is unidimensional and clearly understood by participants (Wanous et al., 1997).

Some assumptions were examined before analyzing the research data. Cook distance values evaluated the presence of outliers, showing that there were no outliers in the data set (Maximum Cook distance value = 0.04). Skewness and Kurtosis coefficients within ±1 indicate a distribution close to normal (Tabachnick and Fidell, 2007). Skewness (0.03 ≤ Skewness ≤ 0.71) and Kurtosis (−0.28 ≤ Kurtosis ≤ 0.76) coefficients in the study show a distribution close to normal. Absence of multicollinearity problem requires variance Inflation Factor (VIF) values below 3 (Yurt, 2023). The highest VIF value in the study was 2.33, indicating absence of multicollinearity problem between the variables (O'Brien, 2007).

Pearson correlation coefficients examined the relationships between socio-demographic characteristics, vulnerable narcissism, grudge and suicide probability scores. Multiple linear regression analysis examined the associations between vulnerable narcissism, grudge, socio-demographic characteristics, and suicide probability. Durbin Watson coefficient shows the autocorrelation risk, a coefficient between 1.5 and 2.5 indicating no autocorrelation risk (Hair et al., 2019; Kalayci, 2010). Durbin-Watson coefficient of 2.00 in the present study showed that there was no risk of autocorrelation. The results show that the research data met the required assumptions for multivariate analyses. IBM SPSS 25.0 software was used for analyses.

## Results

### Correlation analysis results

Table 2 shows the Pearson correlation coefficients between socio-demographic characteristics [sex, age, marital status, education level, socio-economic level (SEL), grudge level and number of siblings] and vulnerable narcissism, grudge and suicide probability scores. The table also shows mean and standard deviation values of vulnerable narcissism, grudge and suicide probability scores.

**Table 2:** Pearson correlation coefficients between socio-demographic characteristics, vulnerable narcissism, grudge and suicide probability.

	Variables	1.	2.	3.	4.	5.	6.	7.	8.	9.	10.
**1**.	Vulnerable narcissism	1									
**2**.	Grudge	0.261^**^	1								
**3**.	Suicide probability	0.436^**^	0.396^**^	1							
**4**.	Sex^a^	0.020	0.139^**^	0.104^*^	1						
**5**.	Age	−0.160^**^	−0.250^**^	−0.365^**^	0.044	1					
**6**.	Marital status^b^	−0.128^**^	−0.212^**^	−0.296^**^	−0.017	0.744^**^	1				
**7**.	Education level	−0.080	−0.017	−0.105^*^	−0.014	0.031	0.034	1			
**8**.	SEL	0.039	−0.019	−0.197^**^	−0.076	0.062	0.083	−0.050	1		
**9**.	Grudge level	−0.022	−0.153^**^	−0.134^**^	0.087	0.126^**^	0.071	−0.069	0.039	1	
**10**.	Number of siblings	0.035	−0.067	0.008	0.017	0.116^**^	0.038	−0.043	−0.085	0.084	1
	Mean	22.96	48.44	70.69		27.57		2.94	1.94	5.80	2.58
	Std. deviation	4.88	15.62	14.85		10.32		0.51	0.46	1.97	1.81

^**^p < 0.01; ^*^p < 0.05; *N*, 500; ^a^0, Female; 1, Male; ^b^0, Single; 1, Married.

Table 2 shows a positive and significant correlation between vulnerable narcissism and grudge (*r* = 0.261, *p* < 0.001) and between vulnerable narcissism and suicide probability (*r* = 0.436, *p* < 0.001). There was a positive and significant correlation between grudge and suicide probability (*r* = 0.396, *p* < 0.001). Some socio-demographic characteristics such as age, marital status and grudge levels and vulnerable narcissism, grudge and suicide probability were significantly correlated.

### Regression analysis results

The multiple linear regression analysis examined the relationships between fragile narcissism, anger, sociodemographic characteristics, and the probability of suicide. The results of correlation analysis conducted in the previous step led to the creation of regression equation. Regression analysis did not include number of siblings since it did not have a significant correlation with suicide probability, which was the dependent variable of the study. Table 3 shows the results found.

**Table 3:** Regression analysis results.

Variable	*B*	SH	β	*T*	*P*
**(Constant)**	66.83	5.60		11.94	0.00^***^
**Sex** ^**a**^	2.21	1.23	0.07	1.81	0.07
**Age**	−0.33	0.08	−0.23	−4.17	0.00^***^
**Marital status** ^**b**^	−0.34	1.43	−0.01	−0.24	0.81
**Education level**	−2.33	1.04	−0.08	−2.24	0.03^*^
**SEL**	−6.09	1.16	−0.19	−5.24	0.00^***^
**Level of religiousness**	−0.50	0.27	−0.07	−1.83	0.07
**Vulnerable narcissism**	1.03	0.11	0.34	9.12	0.00^**^
**Grudge**	0.21	0.04	0.22	5.83	0.00^***^

*R*, 0.619; *R*^2^, 0.383; *F*_(8, 499)_, 38.12; *p* < 0.001; ^***^p < 0.001; ^**^p < 0.01; ^*^p < 0.05; ^a^0, Female; 1, Male; ^b^0, Single; 1, Married; Dependent variable, Suicide probability.

Table 3 presents the results of multiple linear regression analysis conducted to examine the extent to which vulnerable narcissism, grudge, and socio-demographic characteristics (sex, age, marital status, education level, SEL, and level of religiousness) are associated with suicide probability. Regression model shows that independent variables predict suicide probability significantly [*R* = 0.619, *R*^2^ = 0.383, *F*_(8, 499)_ = 38.12, *p* < 0.0001]. Independent variables explained 38% of the change in suicide probability. Vulnerable narcissism (β = 0.34, *p* < 0.001) and grudge (β = 0.22, *p* < 0.001) were positive predictors of suicide probability. Age (β = −0.23, *p* < 0.001), education level (β = −0.08, *p* = 0.003) and SEL (β = −0.19, *p* < 0.001) were significant variables that predicted suicide probability negatively. These results show that having high levels of vulnerable narcissism and grudge is an indicator of high levels of suicide probability and certain socio-demographic characteristics can affect this relationship.

## Discussion and conclusion

### Correlations between vulnerable narcissism and suicide probability

The results of this study show a positive correlation between vulnerable narcissism and suicide probability. In other words, suicide probability increases as the level of vulnerable narcissism increases. High approval and admiration needs are characteristics of narcissism. Studies conducted show that individuals tend to show hostile reactions when they cannot obtain this (Caligor et al., 2015). It can be said that this anger can be directed at themselves or others. Especially in vulnerable narcissism subtype, the failure to meet the need for approval and administration can cause more destructive consequences and suicide may be felt as a way out after a destructive humiliation. Ronningstam et al. (2018) reported high risk for suicide for patients with narcissistic personality disorder and stated that these suicides could occur as fatal attempts without warning. In addition to self-centredness, interpersonal insensitivity, attention-seeking and competitive behaviors in narcissistic individuals, situations such as self-criticism, insecurity, shame and fear can lead to a hidden inner anguish and this anguish can trigger suicidal behavior, sometimes for self-protection and sometimes for escape, due to the feelings of hopelessness and helplessness it causes (Hendin et al., 2007). Chu et al. (2017) reported narcissistic traits and suicide to be significantly correlated. It can be seen that the results of studies conducted in literature are parallel to the results of the present study.

### Correlations between grudge and suicide probability

The results are indicative of a positive significant correlation between grudge and suicide probability and they show that grudge is a predictor of suicide probability. Weiss (1969) reported that grudge was a significant cause of suicide in some types of patients and the motivation in some suicides was to punish family members/loved ones like some kind of revenge. In the literature, grudge is used as the opposite of forgiving, and it is defined as not tolerating a mistake, not forgetting and bearing a grudge. Most of the studies in the literature focus on forgiveness. From this perspective, the results of the present study are in parallel with the literature. Quintana-Orts and Rey (2018) concluded that forgiveness was correlated with suicidal ideation and behaviors and as the level of forgiveness increased, suicidal ideation and behaviors decreased. Another study comparing patients with and without suicide attempt concluded that the scores of individuals with a past suicide attempt were significantly lower than the scores of individuals with no suicide attempt in terms of both self-forgiveness, forgiveness of others and the belief that they would be forgiven (Sansone et al., 2011).

### Demographic variables and suicide probability

The results showed no significant association between sex and suicide probability. The literature shows different results in this regard. While there are studies reporting that there is no significant difference between women and men in terms of suicide risk (Freeman et al., 2017; Rapeli and Botega, 2005), there are also studies reporting that women are more disadvantageous in terms of suicide (Branco et al., 2016; Janghorbani and ve Sharifirad, 2005) and they are in risk in terms of both suicide ideation (Sathian et al., 2015) and suicidal intent (Yamauchi et al., 2013). Eskin (2012) concluded that suicidal ideation and attempts were more common in women, while completed suicide was more common in men. However, Suominen et al. (2004) concluded that being a man was a risk factor for suicide. In addition, there are also studies which showed that suicide frequency (Alptekin and Duyan, 2019; Eskin, 2012); suicide risk (Fukuchi et al., 2013; Masocco et al., 2010) and suicide rates (Alvaro-Meca et al., 2013) were higher in men when compared with women. It can be said that examining the sex variable separately in terms of suicide ideation, suicidal intent, suicide attempt and completed suicides will lead to more significant results. This difference in the results related to sex may be due to interaction with other factors and the differences in gender roles and cultural differences in help seeking behaviors may affect gender related results.

Research results show that suicide probability decreases as age increases. There are different results in the literature regarding this. As there are results similar to the results of the present study, there are also results which show that being old increases the risk. Lizardi et al. (2007) reported that suicide risk was higher in young people. There are studies which concluded that advanced age is a risk factor for suicide (Eskin, 2012; Alptekin and Duyan, 2019).

In the present study, marital status is not a significant predictor of suicide probability. The results are different in terms of marital status in the literature. There are studies which show that marital status does not create a significant difference (Sabanciogullari et al., 2015; Sonderman et al., 2014), while there are also studies which show that suicide risk is higher in married individuals when compared with single individuals (Kposowa et al., 2008; Fukuchi et al., 2013). Studies which examined the status of being single, being divorced and widower concluded that divorced individuals (Alptekin and Duyan, 2019; Sook et al., 2018), widowers (Masocco et al., 2008) and single individuals (Almasi et al., 2009) had a higher risk of suicide when compared with married individuals. These results show that marital status is one of the variables which have the most unstable relationship with suicide among social factors (Sook et al., 2018). It is thought that the potential impact of marital status on suicide may show culture specific differences. However, the meaning attributed to marriage and the fact that experiences have become different show that there is a need to examine this issue in depth.

The present study found education level to be a negative predictor of suicide probability. It is reported in literature that suicide rates increase as education level decreases. There are numerous studies with similar results to the present study (Choi et al., 2022). Studies conducted show that low level of education is associated with suicide (Øien-Ødegaard et al., 2021). However, a study conducted in China reported that the variable of education did not have an effect on suicide ideation (He and Lester, 2001).

Another result of the study is that socio economic level is a negative predictor of suicide probability. There are studies which show that suicides and self-destructive behaviors are more common in low socio economic levels (Rehkopf and Buka, 2006). World Health Organization (2014) reported suicide as one of the main causes of death in low and moderate income countries. Socioeconomic level is considered an important factor in finding out suicide risk and low socioeconomic level is reported to be associated with increased suicide risk (Vijayakumar et al., 2005). Literature results support the results of the present study.

Present study showed that the perceived religiousness was not a predictor of suicide probability. The literature reports different results regarding this. There are studies which showed that religiousness, spirituality and the connection with God had a protective role about suicide (Francis and Bance, 2017; Schnabel et al., 2022); not having a religion was a risk in terms of suicide (Anglin et al., 2005) and low level of religiousness (Aydin-Güler, 2015) predicted suicide probability. There are also studies which show that religiousness increases suicide risk (Sisask et al., 2010; Manoranjitham et al., 2010). The differences in the results of studies may be associated with perceptions of God.

The findings of the study can be interpreted within the framework of the Fluid Vulnerability Theory of suicide (Rudd, 2006; Joiner, 2005). This theory posits that suicidal risk is not static but fluctuates over time between relatively stable baseline vulnerability and acute suicidal states triggered by proximal stressors. In this context, the present results suggest that vulnerable narcissism may function as a distal or baseline vulnerability factor, reflecting a more stable personality disposition associated with increased susceptibility to negative self-referential processing and interpersonal sensitivity. On the other hand, grudge may represent a more proximal cognitive-emotional process that could intensify acute psychological distress and contribute to heightened suicide probability in response to interpersonal experiences. From this perspective, socio-demographic variables such as age, education level, and socio-economic level may serve as protective or risk-modulating factors that influence the overall vulnerability profile. Therefore, the present findings support the core assumption of the Fluid Vulnerability Theory that suicidal risk emerges from the dynamic interaction between enduring vulnerabilities and situational triggers rather than from a single static cause.

### Limitations

The present study has some limitations. First, the researchers asked only one question to determine religiousness level (how religious the participants perceived themselves to be). However, this question does not provide opportunity to analyse the beliefs of individuals in depth. There are also studies with results which show how God is perceived in addition to religiousness and this can be considered as a limitation of the study. As discussed by (Mahoney et al. 2001), any single item religiosity measure cannot properly measure the depth and content of individuals' religiousness. Also, single-item measures can confuse the risks and benefits of religion and thus they may fail in capturing the complex effects of religion on suicide. Future studies can use more effective measures that measure religiousness levels more effectively. The mean age of the sample is around 20 s and therefore can be considered as young adults. The low number of participants from the advanced age can be considered a limitation.

In this study, the sample was determined using convenience sampling, a non-probabilistic sampling method. This can be considered a significant factor limiting the generalizability of the research findings. While convenience sampling allows researchers to collect data from participants easily accessible, it can weaken the sample's ability to represent the population (Etikan et al., 2016). In this context, caution should be exercised regarding the generalization of the findings to larger populations. Furthermore, the possibility of homogeneity among individuals in the sample in terms of certain characteristics may limit the validity of the results for different demographic or cultural groups (Bornstein et al., 2013).

One of the significant limitations of this study is the uneven gender distribution in the sample. The fact that the vast majority of participants were women may limit the generalizability of the findings, particularly to the male population. This sample composition may affect the interpretation of the results, given the gender-sensitive nature of some psychological variables (Hyde, 2014). Future research is recommended to achieve a more balanced gender distribution or to construct the sample using gender-stratified sampling methods. Such approaches will contribute to more robust conclusions by increasing the generalizability of the findings.

Another limitation of this study is the homogeneous nature of the sample in terms of education level and socioeconomic status. The fact that the vast majority of participants are university graduates and from a middle socioeconomic background may limit the generalizability of the findings to different education and socioeconomic groups. In future research, including individuals from different education levels and socioeconomic backgrounds will increase the generalizability of the findings and contribute to more inclusive results.

This study has a cross-sectional design, and the findings are limited to revealing relationships between variables and do not allow for causal inferences (Levin, 2006). Furthermore, the fact that data were collected through self-report measurement tools carries the risk of being susceptible to systematic errors such as social desirability and recall bias (Podsakoff et al., 2003). This necessitates careful interpretation of the findings. It is recommended that future research utilize longitudinal designs and integrate different data collection methods.

### Recommendations

Due to the cross-sectional design of the study, the relationships between the variables cannot be interpreted causally. Therefore, the use of longitudinal or experimental designs in future studies will contribute to a more robust understanding of the trends and possible causal mechanisms between the variables.

The fact that the religiosity variable was assessed with a single-item measure carries the risk of not adequately reflecting the multidimensional nature of this construct. Therefore, it is recommended that future research use multidimensional scales with proven psychometric validity and reliability.

The higher representation of female participants compared to male participants in the sample may limit the generalizability of the findings, especially to the male population. Accordingly, it is recommended that future studies ensure a more balanced gender distribution or include gender as a balancing variable in the model.

## Data Availability

The data analyzed in this study is subject to the following licenses/restrictions: the dataset can be shared upon request. Requests to access these datasets should be directed to gulin_celebi@hotmail.com.
